# Clinical and genetic characterization of patients with eye diseases included in the Spanish Rare Diseases Patient Registry

**DOI:** 10.1186/s13023-024-03242-6

**Published:** 2024-06-13

**Authors:** Alberto Lopez-de la Rosa, Juan J. Telleria, Manuel Posada de la Paz, Isabel M. Hermosilla-Gimeno, Miren Agurtzane Rivas, Raúl Gilabert, Rosa M. Coco-Martín

**Affiliations:** 1 Instituto de Oftalmobiología Aplicada (IOBA), Universidad de Valladolid, Campus Miguel Delibes, Paseo de Belén 17, E-47011 Valladolid, Spain; 2 Observatorio Nacional de Enfermedades Raras Oculares (ONERO), 47011 Valladolid, Spain; 3 Instituto de Biomedicina y Genética Molecular (IBGM), Universidad de Valladolid, 47001 Valladolid, Spain; 4 Facultad de Medicina, Universidad de Valladolid, 47001 Valladolid, Spain; 5 Instituto de Investigación de Enfermedades Raras (IIER), Instituto de Salud Carlos III, 28029 Madrid, Spain; 6 RICORS de Enfermedades Inflamatorias, Carlos III Health Institute, 28220 Madrid, Spain

**Keywords:** Clinical, Genetic, Rare diseases, Eye, Ocular, Registry, Spain

## Abstract

**Background:**

The low prevalence of rare diseases poses a significant challenge in advancing their understanding. This study aims to delineate the clinical and genetic characteristics of patients with rare eye diseases (RED) enrolled in the Spanish Rare Diseases Patient Registry.

**Methods:**

A total of 864 patients from the registry database were included. Diseases were categorized into inherited retinal dystrophies (*n*=688); anterior segment diseases (*n*=48); congenital malformations (*n*=27); and syndromic diseases with ocular involvement including muscular (*n*=46), neurological (*n*=34), or metabolic (*n*=13); inflammatory diseases (*n*=4); and tumors (*n*=4). Data on visual acuity (VA) and/or visual field (VF), symptoms and signs, concurrent diseases in syndromic cases, age of onset and at diagnosis, affected genes, disability rating, inability to work and dependency grade recognition were collected.

**Results:**

A mean diagnostic delay of 7 years from symptom onset was observed. Commonly reported symptoms included photophobia, night blindness, and progressive vision loss (≥57% of patients). Cataract was the most prevalent secondary disease (46%), with pseudophakia being the most common ocular surgery (26%). Hearing loss and cardiovascular diseases were the most prevalent concurrent systemic diseases (≥13%). Certificates of disability, incapacity for work, and dependency were held by 87%, 42%, and 19% of patients, respectively. Among the 719 patients with available VA data, 193 (27%) were blind, and 188 (26%) had moderate to severe visual impairment. Over half of the patients (54%) exhibited VF defects, and 216 (25%) had concentric contraction ≤5° or abolished VF. Most had genetic diseases with autosomal recessive (55%), autosomal dominant (30%), X-linked (9%), and mitochondrial (6%) patterns. One patient had mutations in both recessive *USH2A* and dominant *RHO* genes simultaneously. Of the 656 patients (75.7%) who underwent genetic testing, only 461 (70.3%) received a positive result (pathogenic or likely pathogenic mutations explaining the phenotype). We found 62 new gene variants related to RED not previously reported in databases of genetic variants related to specific phenotypes.

**Conclusions:**

This study delineates the clinical and genotypic profiles of RED in Spain. Genetic diseases, particularly retinal disorders, predominate, but a significant proportion of affected patients remain genetically undiagnosed, hindering potential gene therapy endeavors. Despite notable improvements in reducing diagnosis delays, it is still remarkable. RED frequently lead to disability and blindness among young populations.

## Background

In the European Union, a disease is classified as rare if it affects fewer than 5 individuals per 10,000 [1]. Rare eye diseases (RED) are particularly significant due to their potential to cause irreversible vision loss, often leading to low vision or blindness [2, 3]. Indeed, RED stands as one of the primary causes of blindness among working-age individuals in developed nations [4, 5], compounded by the current lack of effective treatments for many of these conditions [6]. Consequently, RED exerts a substantial impact on patients' quality of life and imposes significant economic burdens on both individuals and healthcare and social systems [7, 8].

While most RED manifest as isolated ocular conditions, a notable proportion are part of syndromic presentations, such as Usher syndrome [9]. The clinical phenotype of RED varies widely based on the affected ocular structures; for instance, Axenfeld-Rieger Syndrome predominantly affects the anterior segment, whereas inherited retinal dystrophies (IRD) primarily impact the posterior pole. However, it is noteworthy that some of these diseases exhibit significant phenotypic heterogeneity [10], often resulting in misdiagnoses of clinically similar conditions. Indeed, phenotypic diversity stands as a primary contributor to the delayed diagnosis commonly observed in certain RED cases [11].

Most RED have a genetic etiology and typically follow Mendelian inheritance patterns (autosomal dominant, autosomal recessive, or X-linked), though other inheritance patterns such as mitochondrial heredity also occur [12]. Over the past decade, the integration of next-generation sequencing (NGS) techniques into clinical practice has facilitated the identification of thousands of genes associated with RED [13]. Furthermore, recent strides in gene therapy development offer promising prospects for patients afflicted with genetic eye diseases [14]. Consequently, molecular genetic diagnosis has emerged as a crucial procedure for individuals suffering from genetic eye diseases.

Despite the challenges posed by the low prevalence of rare diseases (RD) for scientific research, registries serve as invaluable tools for expanding knowledge in this field, elucidating epidemiological trends, and amassing representative sample populations otherwise unattainable [15–17]. In Spain, the Rare Diseases Patient Registry (RePER) [18], operated by the Institute of Health Carlos III, records individuals residing in Spain affected by any RD, including eye diseases. In recent years, healthcare stakeholders have empowered patients to enhance healthcare delivery [19]. Within this context, the National Observatory for Rare Eye Diseases (ONERO) [20], comprising 27 patient associations (established in July 2018), collaborates with RePER (agreement in July 2019) to facilitate registration processes for affected individuals. The data collected hold significant value for clinicians, researchers, and patients alike, offering essential insights to inform research endeavors and public health system management. Therefore, the objective of this study is to delineate the clinical and genetic characteristics of individuals with RED who have self-registered in RePER.

## Methods

An observational study was conducted, involving patients affected by RED included in the RePER since its establishment in 2005. The Institute of Health Carlos III (ISCIII) is the principal governmental research institution in Spain dedicated to health research. Within the ISCIII, the Rare Diseases Research Institute has actively participated in numerous significant European initiatives since its inception, including the RePER, which serves as a national registry, housing clinical and diagnostic data certified by physicians. Its operations are under the supervision of the official Ethics Committee of the ISCIII. As previously said, ONERO has collaborated in assisting patients throughout the registration process.

It is important to clarify that patient-contributed data are solicited alongside consent for registration. Thus, applicants are required to submit medical, ophthalmological, and genetic diagnostic reports along with their registration requests. Once submitted, this information undergoes rigorous review by professionals at the Rare Diseases Research Institute to validate the disease diagnosis before its definitive inclusion in the registry.

RED were identified among all RD using the corresponding ORPHA codes, as suggested by the Orphanet database for such conditions [21]. All participants provided signed, written informed consent in accordance with the European Data Protection Regulation. The study protocol adhered to the principles outlined in the Helsinki Declaration of 1964 (last amendment, 2013). Researchers have systematically gathered all pertinent information used in this study from the registry.

### Sample

The RePER database was consulted on November 25, 2022, to identify patients clinically diagnosed with a RED. Over the past 5 years, ONERO has actively encouraged patients with RED listed in this registry to complete a comprehensive questionnaire and, if available, provide clinical and/or genetic reports. Patient-provided data were meticulously reviewed, and individuals who did not provide reliable clinical data for analysis in the present study, despite being included in the registry, were excluded. Additionally, RED were categorized into the following disease groups: anterior segment diseases, congenital malformations, inflammatory diseases, IRD, metabolic diseases, muscular diseases, neurological diseases, and tumors.

### Data collection

The following data from the general questionnaire were collected: age of symptom onset, age at first visit, age at diagnosis, ocular symptoms, presence of other ocular or systemic diseases, history of ocular surgeries, affected family members, attainment of the Disability Degree Certificate (%), recognition of work incapacity and type (temporary, partial permanent invalidity, total permanent invalidity, absolute permanent invalidity, and severe disability) as per Spanish and European laws [22], and acknowledgment of dependence degree and level (moderate, severe, or profound dependence). The Disability Degree Certificate is an administrative document facilitating access to certain rights and benefits reserved for individuals with disabilities. Disability degree is evaluated using a national scale that assesses the individual's limitations and complementary social factors, expressed as a percentage, with disability being recognized from a degree of 33% onwards.

Distance visual acuity (VA) and visual field (VF) for each eye were collected when available from ophthalmological reports. VA was most frequently recorded on the decimal scale; otherwise (e.g., recorded on logMAR units), it was converted to the decimal scale [23]. Subjects were grouped based on the eye with the highest VA, following the visual impairment and blindness definitions outlined by the World Health Organization in the International Classification of Diseases 11 (ICD 11) [24]: no vision impairment (VA ≥ 0.5 on the decimal scale or VA ≥ 20/40 on the Snellen scale), mild impairment (0.5 > VA ≥ 0.3 on the decimal scale or 20/40 > VA ≥ 20/66 on the Snellen scale), moderate impairment (0.3 > VA ≥ 0.1 on the decimal scale or 20/66 > VA ≥ 20/200 on the Snellen scale), severe impairment (0.1 > VA ≥ 0.05 on the decimal scale or 20/200 > VA ≥ 20/400 on the Snellen scale), and blindness (VA < 0.05 on the decimal scale or VA < 20/400 on the Snellen scale).

Patients included in the RePER registry have undergone VF assessments from its inception in 2005 to the present day at various hospitals and healthcare institutions nationwide. Therefore, data were obtained using various perimeters, predominantly of the static type and VF measurements were taken monocularly. However, given the nature of these pathologies, defects were typically symmetrical in both eyes for nearly all cases in which a VF was provided to the registry. Consequently, the data are presented on a per-patient basis. VF defects were categorized into "central and/or paracentral scotoma", "concentric contraction", "abolished", and "other defects" (e.g., annular scotoma). In cases of concentric contraction, subjects were classified based on the eye with the larger preserved VF, as follows: mild VF contraction (between 25° and 10°), moderate VF contraction (between 10° and 5°), and severe VF contraction (less than 5°).

Genes and mutations identified in genetic reports were gathered. Patients included in the RePER registry have undergone genetic diagnostics since its inception in 2005 up to the present day. Thus, those diagnosed before 2013, prior to the widespread adoption of NGS techniques, underwent genetic diagnosis through mutational analysis of specific genes using Sanger sequencing. Therefore, patients were not uniformly studied using the same genetic diagnostic technique.

The pathogenicity of each mutation (classified as pathogenic, likely pathogenic, uncertain significance (VUS), likely benign, or benign) was reassessed for this study by an expert geneticist (JJT) in accordance with the current guidelines of the American College of Medical Genetics and Genomics (ACMG) variant classification [25]. Whenever possible, the pathogenicity was also corroborated with data from the ClinVar and HGMD databases [26]. Pathogenic and likely pathogenic mutations were consolidated as pathogenic for analysis, while benign and likely benign mutations were grouped as benign. Based on the pathogenicity of each mutation and the inheritance pattern of each gene, the genetic result of each patient was categorized as follows: 1) positive (pathogenic mutations explaining the phenotype were detected); 2) inconclusive (a pathogenic mutation was identified in only one allele in an autosomal recessive disease, while no mutation or a VUS was reported in the other allele); 3) uncertain (only VUS were detected); 4) no findings (no mutations associated with the phenotype were found); 5) negative result, a designation reserved for cases in which the mutation of a relative with an identified pathogenic variant was excluded.

### Statistical analysis

Descriptive analysis was conducted using the R statistical package version 4.1.2. Quantitative data are presented as mean ± standard deviation and range, while qualitative data are presented as frequencies and/or percentages.

## Results

The RePER database, as of November 25, 2022, encompassed 1070 patients clinically diagnosed with a RED (Supplemental Material). Prior to the collaboration between ONERO and RePER in July 2019, 329 patients were already registered, whereas thereafter, 738 patients were added. Following meticulous data scrutiny, 203 patients were excluded due to insufficient data for analysis. Of the latter, 132 patients were registered before the collaborative agreement (constituting 40.1% of patients registered up to that point), while 71 patients were registered afterward (constituting 9.6% of patients registered after the agreement).

**Fig. 1:**
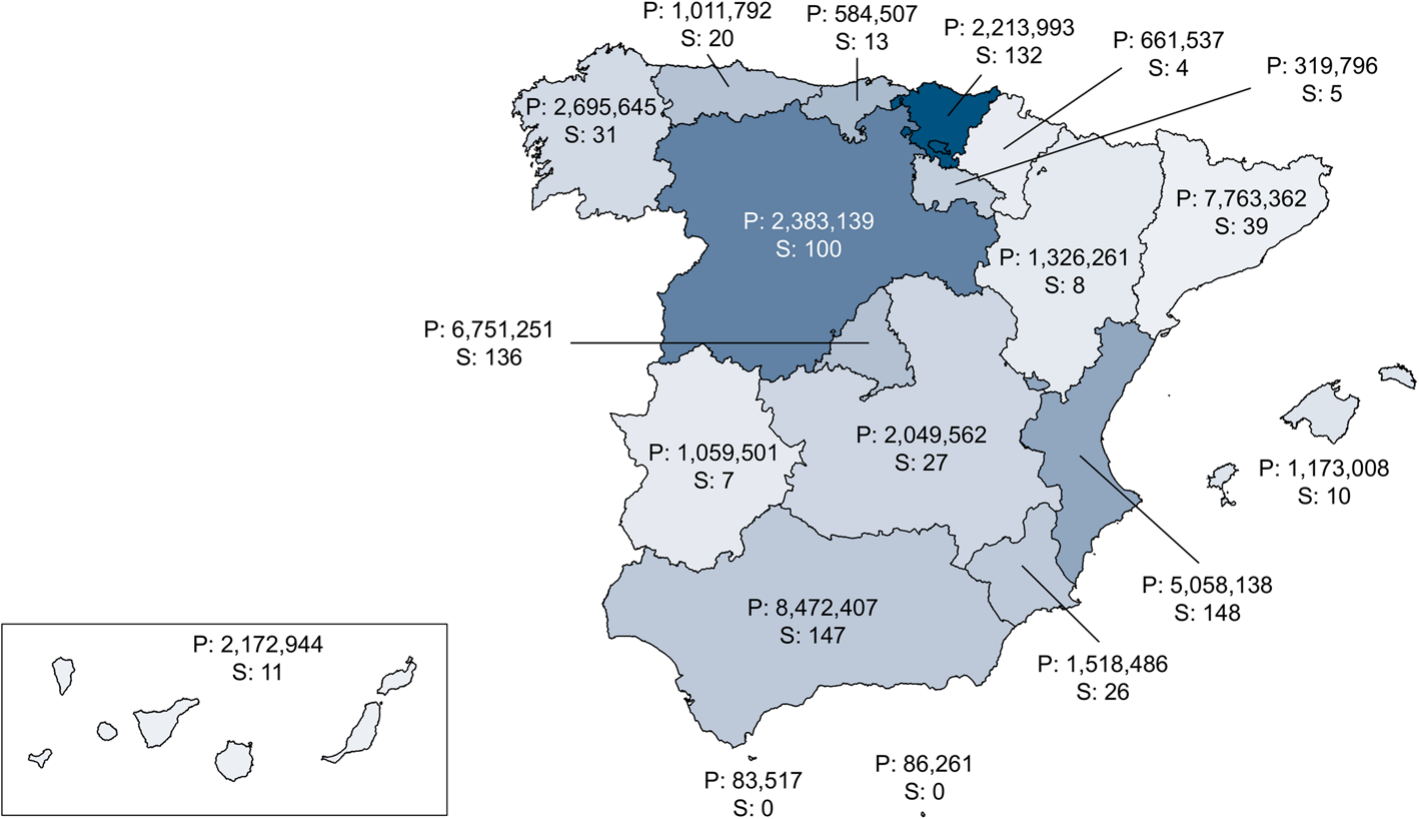
Geographic location of patients included. The color range indicates the percentage (with darker colors representing higher percentages) of the number subjects included (S) as a proportion of the population (P) in each Spanish Autonomous Community, including the two Autonomous Cities of Ceuta and Melilla. Population data was obtained from the Spanish National Statistics Institute (INE), as of January 1, 2021

**Fig. 2:**
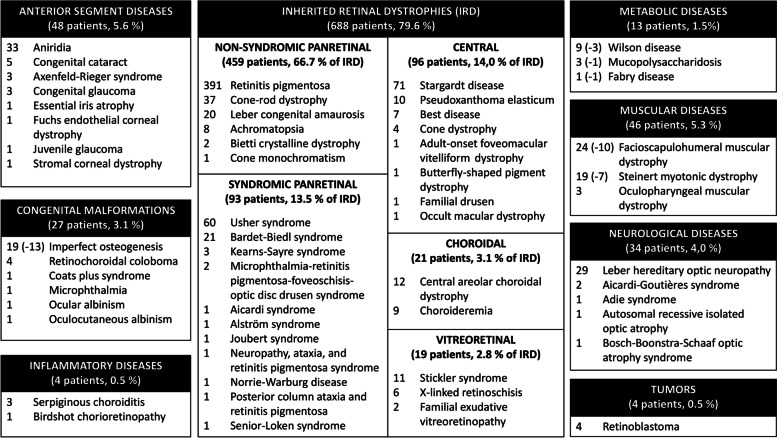
Classification of rare eye diseases and the number of patients included. The number of subjects with a syndromic disease with possible eye involvement but without ocular affectation is indicated as a negative number between brackets

Subsequently, a total of 864 patients with RED, comprising 434 females and 430 males with a mean age of 47.1 ± 18.4 years (range: 3-84), were included in the study. The geographic distribution of included patients is illustrated in Fig. 1. RED were categorized as depicted in Fig. 2.

### Symptoms and age at onset and at diagnosis

The mean age of symptoms onset, age at first clinical visit and age at diagnosis were 17.1±15.7, 20.1±16.5, and 24.3±16.9 years old respectively. There was no delay between the onset of symptoms and the first visit, except in the IRD subgroup (3.3±7.3 years), the muscular diseases subgroup (4.9±10.9 years) and the delay was less significative in the metabolic diseases (0.2±0,4 years).

Regarding the mean diagnosis delay, it was 2.1±10.3 years in anterior segment diseases, 4.7±12.1 years in congenital malformations, 0.5±1.0 years in inflammatory diseases, 8.0±11.5 years in IRD, 2.1±1.6 years in metabolic diseases, 7.8±11.2 years in muscular diseases, 2.7±7.8 years in the neurological diseases group, and there was no delay in tumor diseases. The most prevalent symptoms are shown in Table 1.

**Table 1:** The most prevalent symptoms were reported in the total sample and at each disease group

**Symptoms**	**The total sample (N: 864)**	**Anterior segment (N: 48)**	**Congenital malformations (N: 27)**	**Inflammatory (N: 4)**	**Inherited retinal dystrophies (N: 688)**	**Metabolic (N: 13)**	**Muscular (N: 46)**	**Neurological (N: 34)**	**Tumors (N: 4)**
	**N (%)**	**N (%)**	**N (%)**	**N (%)**	**N (%)**	**N (%)**	**N (%)**	**N (%)**	**N (%)**
Photophobia	519 (60.0)	44 (91.7)	6 (22.2)	1 (25.0)	455 (66.1)	0	2 (4.4)	10 (29.4)	1 (25.0)
Night blindness	510 (59.0)	10 (20.8)	4 (14.8)	3 (75.0)	489 (71.1)	0	0	4 (11.8)	0
Progressive VA reduction	492 (56.9)	21 (43.8)	4 (14.8)	2 (50.0)	449 (65.3)	2 (15.4)	1 (2.2)	13 (38.2)	0
Visual field reduction	403 (46.6)	6 (12.5)	1 (3.7)	1 (25.0)	392 (57.0)	0	0	3 (8.8)	0
Incapacitating glare	363 (42.0)	31 (64.6)	6 (22.2)	3 (75.0)	314 (45.6)	0	0	9 (26.5)	0
Altered color perception	348 (40.3)	5 (10.4)	0	2 (50.0)	320 (46.5)	0	0	21 (61.8)	0
Contrast loss	319 (36.9)	20 (41.7)	1 (3.7)	3 (75.0)	277 (40.3)	0	0	18 (52.9)	0
Sudden VA reduction	162 (18.8)	9 (18.8)	1 (3.7)	1 (25.0)	130 (18.9)	0	0	21 (61.8)	0
Diurnal blindness	140 (16.2)	14 (29.1)	0	1 (25.0)	119 (17.3)	0	0	6 (17.7)	0

*N* The number of subjects. VA: visual acuity

### Other diseases and ocular surgeries

Five hundred and forty-five patients (63.1%) reported suffering from other eye diseases in addition to the reported RED. The most prevalent ones are detailed in Table 2. Two hundred and eighty-six patients (33.1%) reported undergoing ocular surgeries, with the most common procedures outlined in Table 3. Additionally, 408 patients (47.2%) reported having a systemic disease, with the most prevalent ones categorized and presented in Table 4.

**Table 2:** The most prevalent secondary eye diseases/features in the total sample and each disease group

**Secondary eye disease/feature**	**The total sample (N: 864)**	**Anterior segment (N: 48)**	**Congenital malformations (N: 27)**	**Inflammatory (N: 4)**	**Inherited retinal dystrophies (N: 688)**	**Metabolic (N: 13)**	**Muscular (N: 46)**	**Neurological (N: 34)**	**Tumors (N: 4)**
	**N (%)**	**N (%)**	**N (%)**	**N (%)**	**N (%)**	**N (%)**	**N (%)**	**N (%)**	**N (%)**
Cataract	396 (45.8)	36 (75.0)	4 (14.8)	4 (100)	336 (48.8)	1 (7.7)	13 (28.3)	1 (2.9)	1 (25.0)
Nystagmus	81 (9.4)	28 (58.3)	5 (18.5)	0	48 (7.0)	0	0	0	0
Strabismus	71 (8.2)	15 (31.3)	6 (22.2)	0	44 (6.4)	1 (7.7)	2 (4.4)	3 (8.8)	0
Dry eye	51 (5.9)	17 (35.4)	1 (3.7)	0	20 (2.9)	0	12 (26.1)	1 (2.9)	0
Glaucoma	40 (4.6)	18 (37.5)	0	0	19 (2.8)	0	1 (2.2)	2 (5.9)	0
Epiretinal membrane	28 (3.2)	0	0	0	28 (4.1)	0	0	0	0
Corneal alterations	23 (2.7)	14 (29.2)	2 (7.4)	0	7 (1.0)	0	0	0	0
Retinal detachment	16 (1.9)	2 (4.2)	0	0	14 (2.0)	0	0	0	0
Macular hole	15 (1.7)	1 (2.0)	0	0	14 (2.0)	0	0	0	0
Iris alterations	14 (1.6)	11 (22.9)	2 (7.4)	0	0	0	0	1 (2.9)	0

*N* The number of subjects.

**Table 3:** The most common ocular surgeries in the total sample and each disease group

**Ocular surgery**	**The total sample (N: 864)**	**Anterior segment (N: 48)**	**Congenital malformations (N: 27)**	**Inflammatory (N: 4)**	**Inherited retinal dystrophies (N: 688)**	**Metabolic (N: 13)**	**Muscular (N: 46)**	**Neurological (N: 34)**	**Tumors (N: 4)**
	**N (%)**	**N (%)**	**N (%)**	**N (%)**	**N (%)**	**N (%)**	**N (%)**	**N (%)**	**N (%)**
Cataract	224 (25.9)	24 (50.0)	4 (14.8)	0	186 (27.0)	0	9 (19.6)	1 (2.9)	0
Vitrectomy	33 (3.8)	8 (16.7)	1 (3.7)	0	24 (3.5)	0	0	0	0
Refractive	26 (3.0)	1 (2.1)	0	0	23 (3.4)	0	2 (4.4)	0	0
Glaucoma	27 (3.1)	16 (33.3)	0	0	10 (1.5)	0	0	1 (2.9)	0
Strabismus	17 (2.0)	4 (8.3)	2 (7.4)	0	10 (1.5)	0	0	1 (2.9)	0
Keratoplasty	8 (0.9)	6 (12.5)	0	0	2 (0.3)	0	0	0	0
Enucleation/evisceration	6 (0.7)	2 (4.2)	0	0	0	0	0	0	4 (100)

*N* The number of subjects

**Table 4:** The most prevalent systemic diseases/features in the total sample and each disease group

**Systemic disease/feature**	**The total sample (N: 864)**	**Anterior segment (N: 48)**	**Congenital malformations (N: 27)**	**Inflammatory (N: 4)**	**Inherited retinal dystrophies (N: 688)**	**Metabolic (N: 13)**	**Muscular (N: 46)**	**Neurological (N: 34)**	**Tumors (N: 4)**
	**N (%)**	**N (%)**	**N (%)**	**N (%)**	**N (%)**	**N (%)**	**N (%)**	**N (%)**	**N (%)**
Hearing loss	128 (14.8)	2 (4.2)	3 (11.1)	1 (25.0)	111 (16.1)	3 (23.1)	2 (4.4)	4 (11.8)	2 (50.0)
Cardiovascular	109 (12.6)	5 (10.4)	4 (14.8)	0	72 (10.5)	3 (23.1)	19 (41.3)	6 (17.7)	0
Endocrine	77 (8.9)	2 (4.2)	4 (14.8)	0	63 (9.2)	2 (15.4)	6 (13.0)	0	0
Neuromuscular	58 (6.7)	2 (4.2)	1 (3.7)	2 (50.0)	20 (2.9)	2 (15.4)	29 (63.0)	2 (5.9)	0
Respiratory	52 (6.0)	2 (4.2)	2 (7.4)	0	33 (4.8)	2 (15.4)	11 (23.9)	2 (5.9)	0
Neurologic	47 (5.4)	2 (4.2)	1 (3.7)	0	33 (4.8)	5 (38.5)	2 (4.4)	4 (11.8)	0
Obesity	35 (4.1)	2 (4.2)	0	0	31 (4.5)	0	1 (2.2)	1 (2.9)	0
Diabetes	35 (4.1)	3 (6.3)	2 (7.4)	0	26 (3.8)	0	3 (6.5)	1 (2.9)	0
Autoimmune	27 (3.1)	0	2 (7.4)	1 (25.0)	23 (3.3)	1 (7.7)	0	0	0
Congenital malformations of hands or fingers	26 (3.0)	0	1 (3.7)	0	23 (3.3)	2 (15.4)	0	0	0

*N* The number of subjects

### Certificates of disability, incapacity for work, and dependence

Seven hundred and forty-eight (86.6%) possessed a certificate of disability, with a mean disability degree of 77.4±8.8%. Among these, 44 patients from the anterior segment disease group had a degree of 76.6±10.2%, 22 from the congenital malformation group had 66.8±19.6%, 3 patients with inflammatory diseases had 79.7±6.8%, 602 patients with IRD had 74.8±12.9%, 10 patients with a metabolic disease had 63.7±16.7%, 37 patients with a muscular disease exhibited 63.2±17.3%, 28 patients with neurological diseases had 78.4±7.4%, and 2 patients affected by tumors had a degree of disability of 51.0±25.5%.

**Fig. 3:**
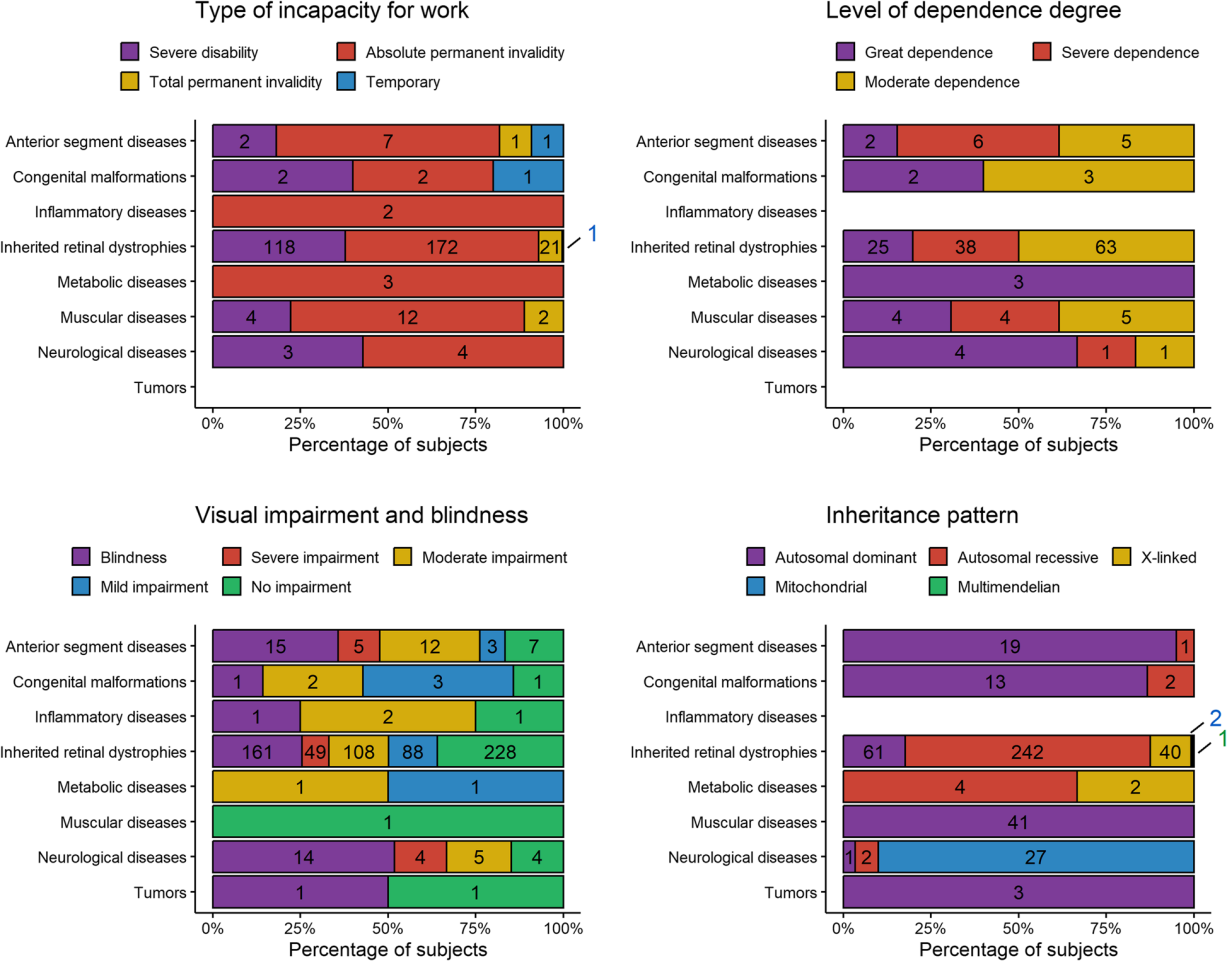
Distributions of type of incapacity for work (top, left), level of dependence degree (top, right), visual impairment and blindness (bottom, left), and inheritance pattern (bottom, right) per disease group. The number in the bars indicate the number of subjects

According to Spanish and European laws, 359 patients (41.6%) were recognized as having incapacity for work: 3 patients (0.8%) had temporary incapacity, 24 patients (6.7%) had total permanent incapacity, 203 patients (56.4%) had absolute permanent incapacity, and 129 patients (36.0%) had severe disability. The types of incapacity for work for each group are detailed in Fig. 3.

One hundred and sixty-six patients (19.2%) had some degree of recognized dependence, categorized as moderate for 77 patients (46.4%), severe for 49 patients (29.5%), and great dependence for 40 patients (24.1%). The levels of dependence degree for each subgroup are illustrated in Fig. 3.

### Visual acuity and visual field

VA data were available for 719 patients (83.2%). Of these, 243 patients (33.8%) had no visual impairment, 95 (13.2%) had mild impairment, 130 (18.1%) had moderate impairment, 58 (8.1%) had severe impairment, and 193 (26.8%) were blind. The classification per disease group is depicted in Fig. 3.

VF results were obtained for 465 patients (53.8%). Among these, 19 had no VF defects. Seventy-one patients (8.2%) presented a VF with central and/or paracentral scotoma (58 belonged to the IRD group, 12 to the neurological group and 1 to the inflammatory group). Two-hundred and seventy-four patients (31.7%) had a concentric contraction of the VF, all of them belonging to the IRD group (39 subjects had a mild contraction, 89 a moderate contraction and 146 a severe contraction). Seventy patients (8.1%) had abolished VF (63 belonged to the IRD group, 6 to the neurological group, and 1 to the anterior segment group). Thirty-one patients (3.6%) presented other defects of VF (23 belonged to the IRD group, 5 to the neurological group, 2 to the anterior segment group, and 1 to congenital malformations group).

### Family members affected and genetic diagnosis

Three-hundred and ninety-one patients (45.3%) reported to have at least one family member diagnosed with the same disease. The most frequently affected family members were siblings (260, 30.0%), followed by mother (75, 8.7%), father (54, 6.2%), maternal cousins (49, 5.7%), sons/daughters (47, 5.4%), maternal uncles/aunts (41, 4.7%), paternal cousins (27, 3.1%), paternal uncles/aunts (25, 2.9%), maternal grandfather (22, 2.5%), maternal grandmother (19, 2.2%), paternal grandfather (11, 1.3%) and paternal grandmother (8, 0.9%).

Six hundred and fifty-six patients (75.9%) had a genetic report. Among these, 461 (70.3%) had a positive genetic result, 47 (7.2%) had an inconclusive result, 36 (5.5%) had an uncertain result, 106 (16.2%) had a non-informative result, and 6 (0.9%) could not be genetically characterized due to the reports presenting incompatible or incongruent information.

The inherited pattern of patients with a genetic disease was autosomal dominant (138, 29.9%), autosomal recessive (251, 54.5%), X-linked (42, 9.1%), mitochondrial (29, 6.3%), and one patient had a multimendelian phenotype of syndromic RP due to mutations in the *USH2A* gene with recessive inheritance and the *RHO* gene which is autosomal dominant. The distribution of inheritance patterns per disease group is depicted in Fig. 3. The genes involved in positive results and the most common mutations, are listed in Tables 5 and 6 respectively. We have identified 62 new pathogenic or likely pathogenic variants that have not been previously reported in genetic variant databases associated with specific phenotypes. However, some of these variants are present in the population database Genome Aggregation Database (gnomAD) v4.1.0. In such cases, allele frequencies and their respective reference SNP identification (rsID) numbers are provided in Table 7. Additionally, two variants have been documented in scientific publications: the c.5898G>A variant in ABCA4 (PMID: 25608812), and the c.946G>T variant in Col1A2 (PMID: 30190494).

**Table 5:** Genes involved in positive genetic results

The disease group (N)		Genes causing disease (N)
**Anterior segment diseases (20)**		*PAX6* (15), *CYP1B1* (1), *FOXC1* (1), *PITX2* (1), *TFGBI* (1), chromosomopathy chr6 (1)
**Congenital malformations (15)**		*COL1A1* (9), *COL1A2* (4), *CTC1* (1), *WNT1* (1)
**Inflammatory diseases (0)**		None
**IRD (346)**	**Non-syndromic panretinal (194)**	*RPGR* (21), *USH2A* (21), *RHO* (16), *ABCA4* (10), *SNRNP200* (10), *CERKL* (8), *CRB1* (8), *EYS* (7), *CNGB1* (5), *PRPF8* (5), *PRPH2* (5), *RP1* (5), *RP2* (5), *CDHR1* (4), *CNGA1* (4), *CNGB3* (4), *NMNAT1* (4), *PRPF31* (4), *RDH12* (4), *AIPL1* (3), *FAM161A* (3), *NR2E3* (3), *PDE6A* (3), *PROM1* (3), *CEP78* (2), *CNGA3* (2), *CYP4V2* (2), *PRPF3* (2), *RPE65* (2), *ACBD5* (1), *ARSG* (1), *C1QTNF5* (1), *CEP290* (1), *CFAP410* (1), *CHM* (1), *CLN3* (1), *GUCY2D* (1), *IMPDH1* (1), *IPMG2* (1), *KCNV2* (1), *MAK* (1), *MERTK* (1), *OPN1LW* (1), *PCARE* (1), *PDE6B* (1), *SAMD11* (1), *SPATA7* (1) , *TULP1* (1)
	**Syndromic panretinal (65)**	*USH2A* (33), *BBS1* (8), *BBS2* (3), *BBS5* (3), *CDH23* (3), *BBS10* (2), *MFRP* (2), *MYO7A* (2), *USH1C* (2), mtDNA deletion (2), *ADGRV1* (1), *ALMS1* (1), *FLVCR1* (1), *TMEM67* (1), *USH2A*+*RHO* (1)
	**Central (63)**	*ABCA4* (55), *BEST1* (4), *ABCC6* (3), *PROM1* (1)
	**Choroidal (12)**	*CHM* (7), *PRPH2* (5)
	**Vitreoretinal (12)**	*COL2A1* (6), *RS1* (5), *COL11A1* (1)
**Metabolic diseases (6)**		*ATP7B* (2), *GALNS* (1), *GLA* (1), *IDS* (1), *SGSH* (1)
**Muscular diseases (41)**		*DUX4* (22), *DMPK* (16), *PABPN1* (3)
**Neurological diseases (30)**		*MT-ND1* (13), *MT-ND4* (11), *MT-ND6* (2), *RNASEH2A* (2), *MT-ND5* (1), *NR2F1* (1)
**Tumors (3)**		*RB1* (3)

*IRD* Inherited retinal dystrophies, *N* number of subjects

**Table 6:** Mutations most frequently identified in genetic reports.

**Alleles**	**Patients**	**Mutation**	**Gene**	**Predicted protein**	**GnomAD frequency**	**Inheritance pattern**	**Associated disease/s**
29	28	c.2276G>T	*USH2A*	p.Cys759Phe (P)	0.002	Autosomal recessive	Retinitis pigmentosa / Usher syndrome
24	20	c.3386G>T	*ABCA4*	p.Arg1129Leu (LP)	0.001	Autosomal recessive	Stargardt disease
22	22	D4Z4 locus partial deletion	*DUX4*	3.4kb units deletion (P)	0.0001	Autosomal dominant	Facioscapulohumeral muscular dystrophy
20	17	c.2299del	*USH2A*	p.Glu767Serfs19* (P)	0.0002	Autosomal recessive	Retinitis pigmentosa / Usher syndrome
16	16	CTG repeat expansion	*DMPK*	3’ non-coding (P)	0.00002	Autosomal dominant	Steinert myotonic dystrophy
12	12	NC_012920.1:m.3460G>A	*MT-ND1*	Mitochondrial (P)		Mitochondrial	Leber hereditary optics neuropathy
12	7	c.1169T>G	*BBS1*	p.Met390Arg (P/LP)	0.002	Autosomal recessive	Bardet-Biedl syndrome
11	11	NC_012920.1:m.11778G>A	*MT-ND4*	Mitochondrial (P)		Mitochondrial	Leber hereditary optics neuropathy
7	7	c.2041C>T	*ABCA4*	p.Arg681Ter (P)	0.0002	Autosomal recessive	Cone-rod dystrophy / Stargardt disease
6	6	c.4457C>T	*ABCA4*	p.Pro1486Leu (P/LP)	0.0001	Autosomal recessive	Cone-rod dystrophy / Stargardt disease
6	6	c.769G>A	*NMNAT1*	p.Glu257Lys (P)	0.001	Autosomal recessive	Retinitis pigmentosa / Leber congenital amaurosis
6	5	c.769C>T	*CERKL*	p.Arg257Ter (P)	0.0003	Autosomal recessive	Retinitis pigmentosa / cone-rod dystrophy
5	5	c.5882G>A	*ABCA4*	p.Gly1961Glu (P/LP)	0.003	Autosomal recessive	Stargardt disease
5	5	c.634C>T	*ABCA4*	p.Arg212Cys (P/LP)	0.0001	Autosomal recessive	Stargardt disease

*P* Pathogenic, *LP* Likely pathogenic, *P/LP* Pathogenic/Likely pathogenic. The Genome Aggregation Database Allele database (gnomAD) v4.1.0 has been used to describe allele frequency

**Table 7:** New variants not previously published in genetic variant databases associated with specific phenotypes

**Alleles**	**Patients**	**Mutation**	**Gene**	**Predicted protein**	**GnomAD frequency**	**Inheritance pattern**	**Associated disease/s**
4	2	c.666dup	*MFRP*	p.Pro223Hisfs15 (LP)		Autosomal recessive	Microphthalmia-retinitis pigmentosa-foveoschisis-optic disc drusen syndrome
2	2	c.2481_2483delinsCT	*ABCA4*	p.Ser827Serfs* (LP)		Autosomal recessive	Stargardt disease
2	2	c.664C>T	*CERKL*	p.Gln222Ter (LP)	6.875e-7 (rs765891262)	Autosomal recessive	Retinitis pigmentosa
2	2	c.2355del	*CNGB3*	p.Ile785IlefsTer? (LP)		Autosomal recessive	Achromatopsia
2	2	c.946G>T	*COL1A2*	p.Gly316Cys (LP)		Autosomal dominant	Imperfect osteogenesis
2	2	c.1106G>A	*COL2A1*	p.Gly316Val (LP)		Autosomal dominant	Stickler syndrome
2	2	c.1313del	*COL2A1*	p.Gly438Alafs182* (LP)		Autosomal dominant	Stickler syndrome
2	2	c.328T>G	*RS1*	p.Cys110Gly (LP)	2.006e-5 (rs770267626)	X-linked	X-linked retinoschisis
2	1	c.2241-¿3634+?dup	*ADGRV1*	p.? (LP)		Autosomal recessive	Usher syndrome
2	1	c.1040C>A	*ARSG*	p.Ala347Asp (LP)	6.572e-6 (rs753990398)	Autosomal recessive	Retinitis pigmentosa
2	1	c.2897del	*ATP7B*	p.Val966Glyfs55 (LP)		Autosomal recessive	Wilson disease
2	1	c.1150C>T	*CNGB1*	p.Glu384* (LP)		Autosomal recessive	Retinitis pigmentosa
2	1	c.278G>A	*CYP4V2*	p.Trp93Ter (LP)	1.368e-6 (rs772955821)	Autosomal recessive	Bietti crystalline dystrophy
2	1	c.1971del	*EYS*	p.Thr657Thrfs5* (LP)		Autosomal recessive	Retinitis pigmentosa
2	1	c.92del	*MERTK*	P.Pro31Leufs32 (LP)		Autosomal recessive	Retinitis pigmentosa
2	1	c.4698_4699del	*RP1*	p.Thr1566Thrfs12* (LP)		Autosomal recessive	Retinitis pigmentosa
1	1	c.378G>A	*ABCA4*	p.Trp126* (P)		Autosomal recessive	Stargardt disease
1	1	c.5044_5088del	*ABCA4*	p.Val1682-Ser1696del (LP)		Autosomal recessive	Stargardt disease
1	1	c.5896G>A	*ABCA4*	p.Glu1966Lys (LP)		Autosomal recessive	Stargardt disease
1	1	c.5898G>A	*ABCA4*	p.Glu1966= (LP)	6.573e-6 (rs1442904666)	Autosomal recessive	Stargardt disease
1	1	c.5938_5943delinsGTGG	*ABCA4*	p.Thr1980Valfs25* (LP)		Autosomal recessive	Stargardt disease
1	1	c.613T>G	*ABCA4*	p.Cys205Gly (LP)		Autosomal recessive	Cone-rod dystrophy
1	1	c.768+2T>G	*ABCA4*	p.? (LP)		Autosomal recessive	Stargardt disease
1	1	c.795-2ª>G	*ABCC6*	p.? (LP)		Autosomal recessive	Pseudoxanthoma elasticum
1	1	c.992T>C	*BBS10*	p.Ala331Val (LP)		Autosomal recessive	Bardet-Biedl syndrome
1	1	c.1894_1895del	*BBS12*	p.Pro632Phefs*7 (LP)		Autosomal recessive	Bardet-Biedl syndrome
1	1	c.1414T>G	*BBS2*	p.Phe472Val (LP)		Autosomal recessive	Retinitis pigmentosa
1	1	c.2916_2917insGCACG	*CDH23*	p.Pro973Alafs18* (LP)		Autosomal recessive	Usher syndrome
1	1	c.8462dup	*CDH23*	p.Leu2821Leufs6* (LP)		Autosomal recessive	Usher syndrome
1	1	c.1868dup	*CDHR1*	p.Asn623Lysfs42* (LP)		Autosomal recessive	Retinitis pigmentosa
1	1	c.1465_1468del	*CERKL*	p.Thr489Leufs10* (LP)		Autosomal recessive	Retinitis pigmentosa
1	1	c.1053del	*CHM*	p.Ile352Ter (LP)		X-linked	Retinitis pigmentosa
1	1	c.659del	*COL2A1*	p.Pro220Leufs88* (LP)		Autosomal dominant	Stickler syndrome
p.1	1	c.732del	*CYP4V2*	p.Trp244Cysfs26 (LP)		Autosomal recessive	Bietti crystalline dystrophy
1	1	c.7361del	*EYS*	p.His2454Profs8* (LP)		Autosomal recessive	Retinitis pigmentosa
1	1	c.1334T>C	*FLVCR1*	p.Leu445Phe (LP)		Autosomal recessive	Posterior column ataxia and retinitis pigmentosa
1	1	c.160dup	*FLVCR1*	p.Arg54Profs36* (LP)		Autosomal recessive	Posterior column ataxia and retinitis pigmentosa
1	1	c.307G>T	*GUCY2D*	p.Glu103Ter (P)		Autosomal recessive	Leber congenital amaurosis
1	1	c.4544_4551delAGATCATGins	*MYO7A*	del framesift (LP)		Autosomal recessive	Usher syndrome
1	1	c.216del	*NMNAT1*	p.Leu72Leufs24 (LP)		Autosomal recessive	Leber congenital amaurosis
1	1	c.1A>T	*PAX6*	p.? (LP)		Autosomal dominant	Aniridia
1	1	c.283_286delinsTTATA	*PAX6*	p.Pro95Leufs2* (LP)		Autosomal dominant	Aniridia
1	1	c.859_862dup	*PAX6*	p.Arg289Serfs9* (LP)		Autosomal dominant	Aniridia
1	1	c.1920+1G>A	*PDE6B*	p.? (P)		Autosomal recessive	Retinitis pigmentosa
1	1	c.784del	*PITX2*	p.Ser262Profs30* (LP)		Autosomal dominant	Axenfeld-Rieger syndrome
1	1	c.1427-2A>G	*PRPF3*	p.? (P)		Autosomal dominant	Retinitis pigmentosa
1	1	c.1272dup	*PRPF31*	p.Glu425Alafs49 (P)		Autosomal dominant	Retinitis pigmentosa
1	1	c.-340-?_1302+?del	*PRPF31*	p.? (P)		Autosomal dominant	Retinitis pigmentosa
1	1	c.(581+1_582-1)_(1041)del	*PRPH2*	p.? (P)		Autosomal dominant	Retinitis pigmentosa
1	1	c.940dup	*RP2*	p.Ile314Aspfs21* (LP)		X-linked	Retinitis pigmentosa
1	1	c.589C>A	*RS1*	p.Arg197Ser (LP)	5.465e-6 (rs281865354)	X-linked	X-linked retinoschisis
1	1	c.638del	*RS1*	p.Met212Argfs+38 (LP)		X-linked	X-linked retinoschisis
1	1	c.426+1G>A	*RTN4IP1*	p.? (LP)		Autosomal recessive	Autosomal recessive isolated optic atrophy
1	1	c.832G>A	*RTN4IP1*	p.Gly278Ser (VUS)		Autosomal recessive	Autosomal recessive isolated optic atrophy
1	1	c.(8681+1_8682-1)_(8845+1_8846-1)del	*USH2A*	p.? (LP)		Autosomal recessive	Usher syndrome
1	1	c.10272_10273dup	*USH2A*	p.Cys3425Phefs3* (LP)		Autosomal recessive	Usher syndrome
1	1	c.12533C>T	*USH2A*	p.Pro4178Leu (VUS)		Autosomal recessive	Usher syndrome
1	1	c.12664_12666delinsGT	*USH2A*	p.Thr4222Valfs35* (LP)		Autosomal recessive	Retinitis pigmentosa
1	1	c.14011G>T	*USH2A*	p.Glu4671Ter (P)		Autosomal recessive	Retinitis pigmentosa
1	1	c.14344-2A>G	*USH2A*	p.? (LP)		Autosomal recessive	Usher syndrome
1	1	c.2296del	*USH2A*	p.Cys766* (LP)		Autosomal recessive	Retinitis pigmentosa
1	1	c.5416A>T	*USH2A*	p.Lys1806Ter (P)		Autosomal recessive	Usher syndrome

*P* Pathogenic, *LP* Likely pathogenic, *VUS* Uncertain significance, *gnomAD* Genome Aggregation Database Allele, *rsID* reference SNP identification number

## Discussion

The low prevalence of RD poses a significant challenge for research efforts. Patient registries dedicated to RD serve as invaluable resources for advancing our understanding of these conditions [15–17]. The observed trends in patient registration and participation before and after the collaboration of ONERO underscore the positive impact of partnerships between patient associations and registries in facilitating patient engagement in research.

In our study, IRD constituted the most significant disease subgroup, accounting for approximately 80% of our total patient cohort. Notably, non-syndromic panretinal disorders, particularly retinitis pigmentosa (RP), were predominant within the IRD category. This prevalence increases when including syndromic cases featuring RP. These findings are consistent with reports from Denmark, where RP accounts for 53% [27] of retinal dystrophies, and France, with 56% [28], indicating that RP is the leading cause of RED in the Spanish population as well.

We observed substantial diagnostic delays across the board, with our cohort experiencing an average delay of seven years, escalating to eight years within the IRD subgroup from symptom onset. In contrast, inflammatory diseases and tumors received diagnoses within a year on average. In line with this, Benito-Lozano et al. [29] report a mean diagnostic time for RD in Spain of 6.2 years, with a median of 7.6 years for ophthalmological diseases, noting a recent decline in these times. Consequently, while RED diagnoses in Spain typically involve some delay, which can impact disease progression, patient and family well-being, and healthcare system costs, the trend towards shorter diagnostic times is been demonstrated, and the delay is not as protracted as with other RD. Importantly, this delay does not significantly differ from findings in the USA (7.6 years) and the UK (5.6 years), as referenced in the Rare Diseases Impact Report [30].

On the other hand, 63.1% of patients reported concurrent ocular pathologies, with cataracts being the most common (45.8%). Although cataract development is frequently linked to aging [31], the average age in our sample was 47.1 years, suggesting additional contributing factors in RED patients. Indeed, it is well known that certain RED are associated with an increased cataract risk, such as RP [32, 33] and aniridia [34]. Consequently, cataract surgery was the most frequently performed procedure in our cohort, with one in four patients having undergone the surgery. Moreover, conditions like nystagmus and strabismus were prevalent, particularly in patients with conditions like cone-rod dystrophies, congenital achromatopsia, or congenital stationary night blindness, where these are anticipated complications due to vision loss and subsequent absence of fixation.

In our cohort, a significant prevalence of systemic pathologies was observed alongside ocular conditions. This prevalence is partly attributable to patients with RED as a component of syndromic disorders [35]. Consistent with previous reports, Usher syndrome emerged as the most common syndromic form of RP [36] and is the predominant genetic cause of concurrent hearing and vision loss. Some affected individuals also exhibit balance disorders and bilateral vestibular areflexia [37]. Notably, hearing loss was the predominant systemic condition, aligning with expectations given that Bardet-Biedl syndrome, the second most frequent syndromic form of RP, similarly involves auditory deficits [38]. These findings underscore that individuals with RED often endure comorbidities beyond visual impairment, necessitating broader clinical attention.

The degree of visual system affectation in RED was assessed by VA and VF data from clinical reports. These metrics facilitated the categorization of patients by the extent of visual impairment. As anticipated, VA and/or VF were significantly compromised across all disease categories. Based on ICD 11 criteria for visual impairment and blindness relating to VA [24], one-quarter of our patients exhibited moderate to severe visual impairment, and another quarter were classified as blind. Additionally, VF assessments revealed that a quarter were visually impaired as having ≤5º preserved. These rates of visual impairment are marginally higher than those reported for IRD in Central European populations, where around 32% of eyes had a VA between 0.5 and 0.1, and 17% had a VA below 0.1 [39]. It is important to note that the referenced study included both eyes per patient, while our study focused on the eye with better VA. Despite this methodological variation, the data indicate that RED typically leads to significant and irreversible vision loss, profoundly affecting patient quality of life [40].

Reflecting the aforementioned visual impairment, most patients in our study possessed a disability certificate, with the average degree of disability exceeding 60% across all disease groups except for tumors. Furthermore, two-fifths of our cohort had been granted incapacity for work, with the vast majority qualifying for either absolute permanent or severe disability. This is in stark contrast to the situation among RP patients in France, where only 19.6% had received a disability allocation, yet 55.4% were classified as disabled workers [8]. The variation between these findings may be attributed to the differences in the countries' legal frameworks. Additionally, approximately 20% of patients were acknowledged to have a degree of dependency, highlighting the debilitating nature of visual impairment in performing essential life activities.

The etiology of RED is predominantly genetic, with nearly half of our patients reporting a family history of the same condition. The importance of genetic diagnosis has surged in recent years, especially due to the advancements in gene therapy. However, a significant portion (24.1%) of our patients with a genetic disorder have not yet undergone genetic testing. Of those who have been tested, approximately two-thirds received a positive genetic diagnosis. The most frequently observed inheritance pattern was autosomal recessive, particularly within the IRD group, which aligns with findings from previous studies [41, 42]. Conversely, autosomal dominant patterns were more prevalent in conditions affecting the anterior segment, congenital malformations, and muscular disease groups. Mitochondrial inheritance was chiefly noted in neurological diseases, largely due to the inclusion of patients with Leber hereditary optic neuropathy [43]. Common genes and mutations associated with IRD, such as *USH2A* (c.2276G>T) and *ABCA4* (c.3386G>T), have been well-documented both globally and in Spain [42]. Most of the mutations have been previously described and in no case have they been reported as part of a complex allele. Furthermore, genotype-phenotype correlation has been evaluated in all cases. However, we cannot rule out that in some cases of autosomal recessive disorder, the two identified variants could be in cis, constituting a single allele as segregation studies have not always been sent by patients. This study also identifies the more frequent genes and 62 variants not previously included in databases of genetic variants related to specific phenotypes, which has potential clinical and research interest, although as previously mentioned 2 of them have been reported in scientific reports [44, 45].

The diagnostic yield of 70% is relatively high, but it's important to note that this figure pertains to those who have received a genetic diagnosis, not the entire sample. In fact, just over half of the total sample (53.4%) have been genetically diagnosed (461 out of 864). It should also be mentioned that registration requires confirmed diagnoses with medical and/or genetic reports, which may skew this percentage. However, recent literature suggests that diagnostic yields for genetic studies in IRD (the most numerous subgroup) range from 52% to 74%, placing our findings at the higher end of the spectrum [46].

The heterogeneity of clinical and genetic data sources, collected from various clinicians at different clinics, may be seen as a limitation of this study. Furthermore, as the registry is focused on individual patient records, we have not accounted for the total number of enrolled families. Some regions, despite having a high population density, such as Catalonia, contributed fewer cases to the registry. The diversity of diseases included, with some represented by only a single patient, may also pose interpretive challenges. This is partly due to the low prevalence of certain conditions. To mitigate data variability, diseases were categorized into groups based on the part of the eye they affect, rather than their etiology. Nonetheless, further research with a larger cohort of patients suffering from these RD is warranted.

## Conclusions

In summary, registries prove to be useful for stakeholders as they permit the delineation of the clinical and genetic landscape of RED. Collaboration with patient associations is a wonderful and useful tool to increase the availability of patient data for research purposes. IRD, particularly Retinitis RP, are the most common rare eye conditions in the Spanish population, as anticipated. The diagnosis of RED is often delayed, although this delay is fortunately decreasing over time and is shorter than that observed for other RD. However, this delay can exacerbate disease progression or lead to suboptimal treatments. The significant visual impairment resulting from RED leads to substantial disability and blindness at young ages, profoundly affecting patients' quality of life. Currently, approximately half of the patients with a genetic disease have not received a definitive diagnosis, limiting their access to emerging gene dependent therapies. Finally, new 62 new variants of interest have been identified.

## Data Availability

Access to data in the registry itself is available to authorized users under conditions governed by the registry data access policy set out on the registry website at https://registroraras.isciii.es/Comun/Inicio.aspx. The data protection policy according to GRDP standards of the RePER registry can be found in: https://www.isciii.es/InformacionCiudadanos/_layouts/15/WopiFrame.aspx?sourcedoc=%7B22A0BEC5-A52D-4375-BEBF-D10AC1C2D133%7D&file=Politica_Privacidad_ISCIII.pdf&action=default. Owing to intellectual property restrictions, the full registry codebook is not publicly available but, is available from the responsible staff of the registry on a reasonable request, subject to certain conditions restricting commercial use.
